# *Porphyromonas gingivalis* Promotes 4-Nitroquinoline-1-Oxide-Induced Oral Carcinogenesis With an Alteration of Fatty Acid Metabolism

**DOI:** 10.3389/fmicb.2018.02081

**Published:** 2018-09-04

**Authors:** Jia-shun Wu, Min Zheng, Mei Zhang, Xin Pang, Li Li, Sha-sha Wang, Xiao Yang, Jing-biao Wu, Ya-jie Tang, Ya-ling Tang, Xin-hua Liang

**Affiliations:** ^1^State Key Laboratory of Oral Diseases, National Clinical Research Center for Oral Diseases, Department of Oral and Maxillofacial Surgery, West China Hospital of Stomatology, Sichuan University, Chengdu, China; ^2^Department of Stomatology, Zhoushan Hospital, Wenzhou Medical University, Zhejiang, China; ^3^Key Laboratory of Fermentation Engineering (Ministry of Education), Hubei Provincial Cooperative Innovation Center of Industrial Fermentation, Hubei Key Laboratory of Industrial Microbiology, Hubei University of Technology, Wuhan, China

**Keywords:** fatty acids, fatty acid synthases, mouse models, oral squamous cell carcinoma, *Porphyromonas gingivalis*, 4-nitroquinoline-1-oxide

## Abstract

Microbiota has been widely considered to play a critical role in human carcinogenesis. Human papilloma virus, hepatitis B and C virus, and *Helicobacter pylori* are implicated in the pathogenesis of cancer of uterine cervix, liver, and stomach, respectively. However, whether *Porphyromonas gingivalis (P. gingivalis)*, a common Gram negative oral bacteria, is associated with oral carcinogenesis still remains unclear and its underlying mechanism needs to be addressed. Here, we established a combined experimental system of 4NQO-induced oral carcinoma model and chronic periodontitis model and investigated the effects of *P. gingivalis* infection on oral carcinogenesis and fatty acid metabolism during oral carcinogenesis. The data showed that in this animal model, *P. gingivalis* infection induced mice periodontitis, increased the tongue lesion size and multiplicity of each mouse and promoted oral cancer development. *P. gingivalis* treatment significantly increased the level of free fatty acids and altered the fatty acid profile in tongue tissues and the serum of mice. And *P. gingivalis* induced the formation of fatty liver of the mice. Besides, immunohistochemical analysis and qRT-PCR showed that the expression of fatty-acid synthase and acetyl-CoA carboxylase 1 were increased in the tongue and liver tissues of 4NQO-treated mice infected with *P. gingivalis*. These results showed that *P. gingivalis* promoted oral carcinogenesis and aggravated disturbance of fatty acid metabolism, indicating a close association among *P. gingivalis*, lipid metabolic and oral carcinogenesis.

## Introduction

*Porphyromonas gingivalis*, a common Gram negative oral bacteria, is a major periodontal pathogen. Apart from gingival biofilm, *P. gingivalis* is abundantly present on other sites of oral cavity including tongue dorsum (Faveri et al., 2006). Recently, there is increasing evidence supporting that *P. gingivalis* is involved in the development and progression of several types of gastrointestinal tract cancers, including colon, pancreatic, and oral cancers (Tezal et al., 2007; Ahn et al., 2012; Michaud, 2013; Michaud et al., 2013). *P. gingivalis* can penetrate and invade various epithelial cells and attach to different gingival carcinoma cell lines (Hirose et al., 1996). Moreover, there are similar symptoms between oral cancer and periodontal lesions, such as swelling, bleeding, tooth mobility, deep periodontal pocket, and bone destruction (Fitzpatrick and Katz, 2010; Yang et al., 2018). It has been reported that each millimeter of alveolar bone loss of chronic periodontitis is associated with a 5.23-fold increase in the risk of tongue cancer, and *P. gingivalis* infection was an independent prognostic factor for the overall survival of the patients with orodigestive cancer (Tezal et al., 2007; Ahn et al., 2012). *P. gingivalis* has been shown to inhibit the apoptosis of epithelial cells and induce the alteration of epithelial cells to neoplastic forms by promoting cell proliferation and survival (Katz et al., 2011). These indicated that *P. gingivalis* might be an important etiological factor of OSCC. However, the role and its molecular mechanism of *P. gingivalis* in the development and progression of OSCC still remain unclear.

Oral squamous cell carcinoma is one of the most common cancers with a high propensity for local recurrence and metastasis, most commonly in the neck lymph nodes (Petersen, 2009). Risk factors for OSCC include tobacco, alcohol, human papillomavirus, and poor hygiene (Leemans et al., 2011; McCormick et al., 2015). Despite continuing advances in therapy, the 5-year survival rate of OSCC patients has still remained at approximately 50% (Torre et al., 2015). Cellular metabolism alteration is one of the hallmarks of cancer, and FA metabolism in OSCC have increasingly attracted interests of researchers (Warburg, 1956; Currie et al., 2013). The FA profile was showed to be significantly different between OSCC and normal tissues, and the levels of eicosapentaenoic acid and docosahexaenoic acid can be regarded as diagnostic and prognostic biomarkers (Askari et al., 2015). FA binding protein 4 and 5 was overexpressed in OSCC, and FABP4-specific siRNA inhibited the cell growth of OSCC through the MAPK pathway, whereas overexpression of FABP5 enhanced cell proliferation and invasiveness by upregulating the expression of MMP-9 (Fang et al., 2010; Lee et al., 2014). These indicated that FA metabolism products and related signaling pathway may be prognostic biomarkers and therapeutic targets for oral cancer (Askari et al., 2015; Harjes et al., 2016).

Importantly, chronic periodontitis and *P. gingivalis* were showed to be associated with lipid metabolic diseases, including atherosclerosis and fatty liver (Hayashi et al., 2011; Li et al., 2013). New Zealand White rabbits with periodontitis induced by *P. gingivalis* had more extensive accumulations of lipids in the aorta than nonperiodontitis animals (Jain et al., 2003). Moreover, the links between *P. gingivalis* and hepatic steatosis were observed in an experimental periodontitis mice model (Nakajima et al., 2016). Although the effects of *P. gingivalis* on lipid metabolism were recognized, whether *P. gingivalis* has similar influences on FA metabolism during oral cancer development remains unclear.

Here, to investigate the role of *P. gingivalis* in oral carcinogenesis and its association with FFA metabolism, we used 4NQO and *P. gingivalis* to establish a mice model of chronic infection-associated oral tumorigenesis. GC–MS was applied to examine the FA profile in the serum and tongue tissues. In addition, IHC and quantitative reverse transcription polymerase chain reaction (qRT-PCR) were used to detect the expression of FASN and ACC1 to investigate the change of *de novo* FA synthesis pathways. This will provide some helpful evidence to support address whether there is a close association among *P. gingivalis*, lipid metabolic and oral carcinogenesis.

## Materials and Methods

### Animals and Experimental Design

**FIGURE 1:**
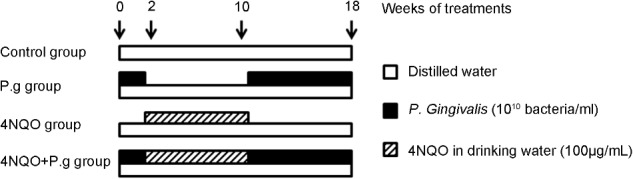
Diagram of the experimental protocol. Oral administration of *P. gingivalis* was given to the mice in P.g and 4NQO + P.g group in the period of 0–2 weeks and 10–18 weeks. And the mice in 4NQO and 4NQO + P.g group were exposed to 4NQO treatment in the period of 2–10 weeks. The control mice were treated with vehicle alone.

A total of 65, 6- to 8-week-old female wild-type C57BL/6 mice (Dashuo Co., Ltd., Chengdu, China) matched for age and weight were used. The animals were housed in State Key Laboratory of Oral Diseases, West China Hospital of Stomatology (Sichuan University) at standard laboratory conditions. After a week of acclimation, mice were randomly divided into four experimental groups; control group (*n* = 10), P.g group (*n* = 15), 4NQO group (*n* = 20), and *P. gingivalis* + 4NQO group (*n* = 20). A stock solution of 4NQO (Sigma, St. Louis, MO, United States) was prepared at 0.5 mg/mL and added to the drinking water to obtain working concentration of 100 μg/mL. Mice in 4NQO and 4NQO + P.g group were given 100 μg/mL 4NQO for 8 weeks, and then switched to distilled water for 10 weeks. The animals in P.g and 4NQO + P.g group was repeatedly infected with *P. gingivalis* (strain 381) (200 μL of 10^10^ bacteria/mL) that suspended in 2% carboxymethylcellulose, as described previously (Currie et al., 2013). Mice in these groups were given *P. gingivalis* infection (three times/week) for 2 weeks prior to 4NQO administration in 4NQO + P.g group; after 8-weeks 4NQO treatment, infection was continued for these groups (twice a week) for 10 weeks. Mice in control group didn’t treat with 4NQO or P.g Diagram of the experimental protocol was shown in **Figure 1**.

At the end of the experimental period, mice were anesthetized using isoflurane, and blood, tongue, maxillary, and liver tissues were collected. Blood samples in vacutainer vials containing heparin were obtained and immediately centrifuged at 3,000 ×*g* for 10 min and separated serum samples were then stored at −80°C before analysis. Tongue were collected and then longitudinally bisected. One part of each tongue tissue was fixed in 10% buffered formalin immediately and embedded with paraffin. The other part was immediately snap-frozen and stored at −80°C for FFAs analysis or qRT-PCR. The maxillary and liver tissues from each group were collected and fixed in 10% buffered formalin for 24 h. The maxillary was decalcified in 15% EDTA and then embedded in paraffin after dehydration using a graded ethanol series.

### Ethics Statement

All animal experiments were conducted according to the procedures approved by the Subcommittee on Research and Animal Care (SRAC) of Sichuan University.

### Detection of *P. gingivalis* in Mouse Tongues by PCR

After 24 h of *P. gingivalis* administration, a sterile cotton swab was used to collect samples by rubbing against the tongue surface of each group mice for 30 s. The collected samples were vortexed in 200 μL of sterile ultrapure water for 5 s and then boiled for 10 min, following by a centrifugation at 12,000 *g* for 10 min to collect the supernatant (DNA template). DNA amplification was performed by KOD-Plus High fidelity PCR polymerase (Toyobo Co., Ltd. Life Science Department, Osaka Japan) according to the manufacturers’ instructions and with primers specific for *P. gingivalis* 16S rRNA. PCR conditions were as follows: an initial denaturation step at 95°C for 10 min, 30 cycles of denaturation at 94°C for 15 s, hybridization at 61°C for 30 s, and elongation at 72°C for 30 s. The primers used were as follows: forward: 5′-GT GAGGTAACGGCTCAC CAA-3′, reverse: 5′-GTATCGCCCGTTATTCCCGT-3′.

### Histopathological Analysis

A total of 5-μm sections of tongue, gingival, and liver tissues from different groups were processed for HE staining for histopathology. Histopathological examination was performed by an experienced oral pathologist in a blind manner basically according to the criteria described by Kramer et al. (1978).

### Immunohistochemical Analysis

Tongue sections at 5-μm were deparaffinized and rehydrated in a graded ethanol series to distilled water. For antigen retrieval, slides were immersed in 0.01 M sodium citrate buffer, and heated in a steamer for 30 min. After inhibition of endogenous peroxidases by 3% hydrogen peroxide and blocking with goat serum albumin, sections were incubated overnight at 4°C with anti-FASN antibody (1:100, Proteintech Group, Chicago, IL, United States), anti-ACC1 antibody (1:100, Proteintech Group, Chicago, IL, United States). The slides were incubated with biotinylated secondary antibody and then with peroxidase–streptavidin (ZSGB-Bio, China), followed by stain of 3,3′-diaminobenzidine tetrahydrocloride (DAB) for 5 min. Finally, nuclei were counterstained with hematoxylin. As negative controls, tissue sections were processed in parallel by incubating with PBS instead of the primary antibody.

The semiquantitative assessment of IHC staining was performed by evaluating the staining intensity and the percentage of positive cells. The intensity was graded as 0 (no staining), 1 (mild staining), 2 (moderate staining), and 3 (strong staining). The percentage of positive cells was evaluated as: 0 (negative), 1 (0–10% positive), 2 (10–30% positive), or 3 (>30% positive).The index of staining was calculated as the percentage of staining multiplied by staining intensity.

### qRT-PCR

Total RNA was extracted from tongue tissues of each experimental group using a mini RNeasy kit (Qiagen). A total of 700 ng of RNA was reverse transcribed using the High-Capacity cDNA kit Reverse Transcription (Applied Biosystems, United States) according to the manufacturer’s instructions. PCR amplification was performed for determinate the expression of FASN and ACC1 using TaqMan chemistry (TaqMan Master Mix Fast, Applied Biosystems, United States). The PCR program included 50°C for 2 min, 95°C for 10 min, 40 cycles at 95°C for 15 s, and 60°C for 1 min. The primers used were as follows: forward: 5′-ATGGGGAAGGTGAAGGTCG-3′ and reverse: 5′-TAAAAGCAGCC CTGGTGSACC-3′ for *GAPDH*; forward: 5′-TCGTGGGCTACAGC ATGGT-3′ and reverse: 5′-GC CCTCTGAAGTCGAAGAAGAA-3′ for *FASN*; forward: 5′-ATCATT GCTCCTCCT GAG CGC-3′ and reverse: 5′-TCTGAAAGCTTTCAGTCTCTA-3′ for *ACC1*. Finally, the expression of each gene was calculated using the 2^−ΔΔCT^ method and GAPDH was used as the internal control (Livak and Schmittgen, 2001).

### Micro-CT Analysis of Alveolar Bone Loss

Maxillary hemi-jaws of each group mice were examined by a micro-CT system (mCT 40, Scanco Medical, Bassersdorf, Switzerland) at a resolution of 12 μm in all three spatial dimensions as described previously (Wilensky et al., 2005). The sagittal plane of the maxillary hemi-jaws was set parallel to the X-ray beam axis.

### Analysis of FFA Composition in Serum and Tongue Tissues

Fatty acid compositions in serum and tongue tissues were measured by GC–MS, as described previously (Gieng et al., 2005; Currie et al., 2013). A total of 50 mg minced tissues fraction or aliquots (200 μL) of serum was mixed with 2 mL of 10% H_2_SO_4_–CH_3_OH and then kept in a water bath of 70°C for 2 h. Then 2 mL of hexane and saturated sodium chloride solution were sequentially added into the solution to obtain the FA methyl esters. Organic phases were collected and dried under N_2_ and samples were dissolved in 100 μL hexane and then analyzed by GC–MS. The relative content of each FA composition was determined by the percentages of its peak areas to total FAs in the GC–MS chromatogram.

### Analysis of FFAs Concentration in Serum and Tongue Tissues

A total of 10 μL serum and 20 mg tongue tissues was taken to measure FFAs concentration using a Free Fatty Acid Quantification Colorimetric Kit (BioVision, Milpitas, CA, United States) according to the manufacturers’ instructions.

### Statistical Analysis

Data were analyzed with the Statistical Package for Social Science version 22.0 (SPSS; Chicago, IL, United States) and GraphPad Prism (version 5.03; San Diego, CA, United States). The quantitative data were expressed as mean ± standard deviations (SD) and were analyzed by one way ANOVA. Tukey test was performed for multiple testing between the two different groups. Differences between proportions were performed using Chi-square test. *P*-values <0.05 were considered statistically significant.

## Results

### General Observations of Animal

**FIGURE 2:**
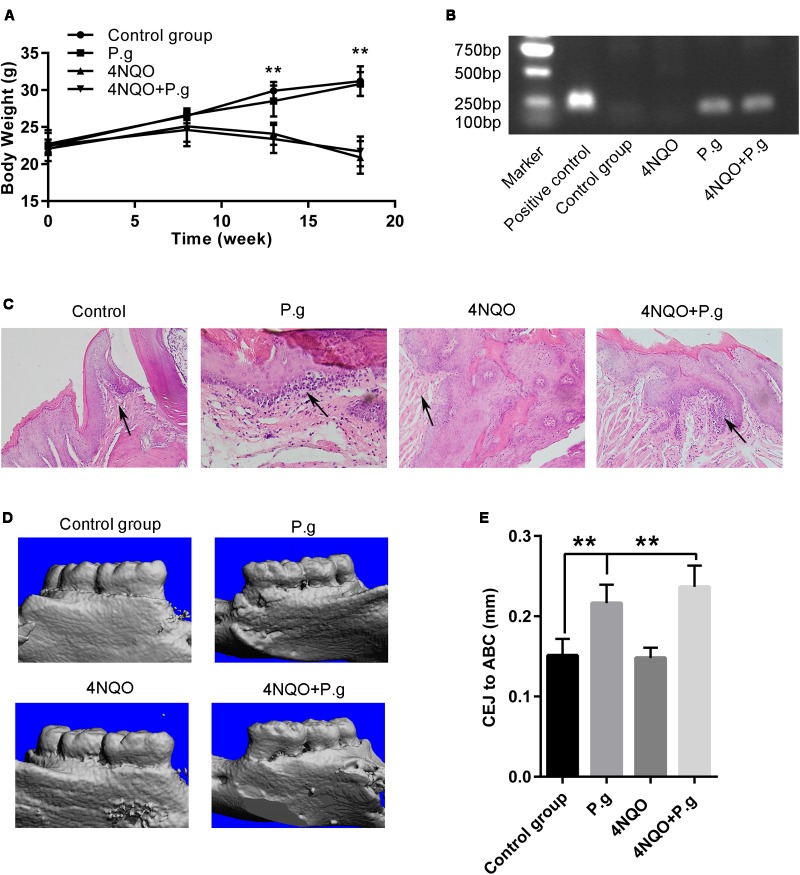
Oral administration of *P. gingivalis* induced periodontitis in the mice. **(A)** The body weights of animals in control, P.g, 4NQO, and 4NQO + P.g group. The data represent the mean ± SEM of 10 animals per group at the indicated times; ^∗∗^*P* < 0.01. **(B)** Representative results of colony PCR for detection of *P. gingivalis* in the mice oral cavity. **(C)** Histochemical analysis of gingival tissues of mice each group. The histological images of gingival tissues in P.g and in 4NQO + P.g group had more inflammatory cell infiltration compared with 4NQO and control group. All images were taken at 400× magnification. **(D)** Representative results of micro-CT analysis of maxillary hemi-jaws in mice from each group. The levels of alveolar bone crest (ABC) in mice of control and 4NQO group were at the cement-enamel junction (CEJ), whereas severe bone absorption was observed in mice of P.g and 4NQO + P.g group. **(E)** The quantitative data of alveolar bone loss was measured as distance from the CEJ to the ABC. The columns represent the mean ± SEM of 10 animals per group; ^∗∗^*P* < 0.001.

To elucidate the role of *P. gingivalis* in OSCC development, a combined experimental system of 4NQO-induced oral carcinoma model and *P. gingivalis-*induced chronic periodontitis model was established as described previously (Binder Gallimidi et al., 2015; Wang et al., 2016). The consumption of water and basal diet per mouse of each group was comparable. The body weights of 4NQO-exposed group were significantly lower than that without 4NQO treatment at the end of 13 and 18 weeks (**Figure 2A**). However, there was no significant difference in body weights among the mice with or without *P. gingivalis* oral administration, which indicated that the mice were tolerant of *P. gingivalis* administration.

### The Mice Periodontitis Induced by *P. gingivalis*

Colony PCR was used to detect the presence of *P. gingivalis* in mouse tongues by oral challenge after 24 h of oral administration. The results showed that *P. gingivalis* was detected both in P.g and 4NQO + P.g group, but neither in control nor in 4NQO group (**Figure 2B**). *P. gingivalis* was detected in the 92% mice of P.g group and 96% in 4NQO + P.g group. Histological examination revealed that gingival tissues in P.g and 4NQO + P.g group had some inflammatory cell infiltration compared with control group. However, inflammatory cell infiltration was hardly observed in 4NQO group (**Figure 2C**). Micro-CT analysis of bone loss was conducted to examine the development of periodontal disease in the model. The mean crestal bone loss in P.g and 4NQO + P.g group was (0.22 ± 0.02) mm and (0.24 ± 0.03) mm, respectively, and was significantly severer than the control mice (0.15 ± 0.02) mm (**Figures 2D,E**). These findings suggested that oral administration of *P. gingivalis* was an effective approach to induce periodontitis of mice, consistent with previous study (Polak et al., 2009; Binder Gallimidi et al., 2015).

### *P. gingivalis* Infection Enhanced Tongue Carcinogenesis in 4NQO-Treated Mice

**FIGURE 3:**
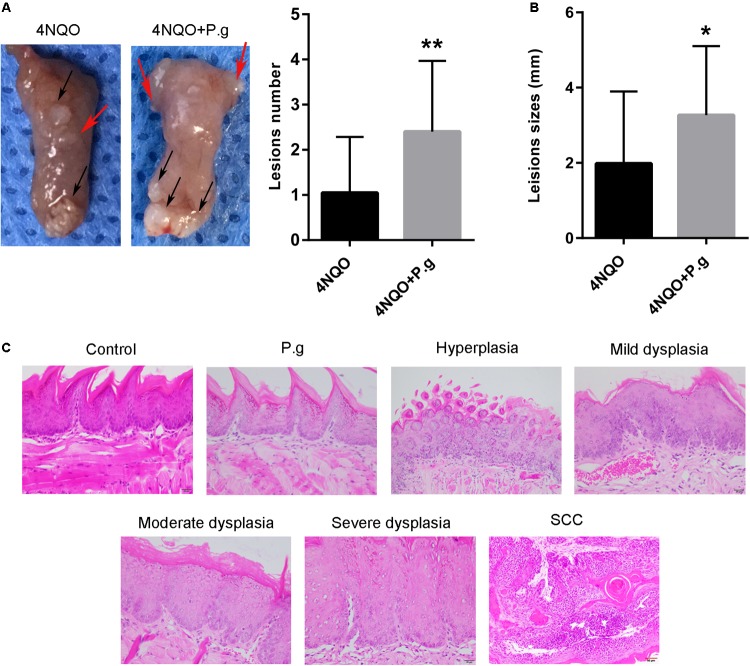
Effect of *P. gingivalis* infection on tongue carcinogenesis in 4NQO-treated mice. **(A)** Representative imagines tongue tissues and the quantitative data of lesion number per mice in 4NQO and 4NQO + P.g group were shown. *Black arrows* indicate tumors; *red arrows* indicate dysplasia. The columns represent the mean ± SEM of 10 animals per group; ^∗∗^*P* < 0.01. **(B)** The average of lesion sizes in mice of 4NQO and 4NQO + P.g group. The columns represent the mean ± SEM of 10 animals per group; ^∗^*P* < 0.05. **(C)** Representative HE sections of pathology, including hyperplasia, mild dysplasia, moderate dysplasia, severe dysplasia, and carcinoma. All images were taken at 400× magnification.

The gross examination of tongue lesions showed no visible lesion in P.g and control group. However, 4NQO-treated mice (4NQO and 4NQO + P.g group) developed obvious precancerous and cancerous lesions. A total of 80% (16/20) mice in 4NQO + P.g group showed oral lesions compared with 55% (11/20) mice in the 4NQO group. In 4NQO group, the tumor lesion was (1.98 ± 1.92) mm on average and the average number of lesion per mouse was (1.1 ± 1.2). However, in 4NQO + P.g group the tumor lesion was (3.27 ± 1.83) mm on average and the average number of lesion per mouse was 2.4 ± 1.6, which showed that P.g contributed to the formation of tongue lesion induced by 4NQO (**Figures 3A,B**).

Then, HE staining showed that 4NQO-treated mice exhibited different stages of oral carcinogenesis compared with normal epithelia in control group. All samples were divided into the following six types: normal epithelium, hyperplasia, mild dysplasia, moderate dysplasia, severe dysplasia, and carcinoma (**Figure 3C**). The results of histopathological analysis of 4NQO and 4NQO + P.g group were summarized in **Table 1**. A total of 65% (13/20) mice in the 4NQO + P.g group developed squamous cell carcinoma compared with 45% (9/20) mice in the 4NQO group. A two-category system (normal/hyperplasia/mild or moderate dysplasia: low risk; severe dysplasia/carcinoma: high risk) was used to analyze the oral cancer risks of mice in the two groups, according to previous studies (Warnakulasuriya et al., 2008; Tang et al., 2009). In the 4NQO + P.g group, 80% of the mice showed a high risk of oral carcinogenesis, compared with 50% of the mice in the 4NQO group (*P* < 0.05). These indicated that *P. gingivalis* infection enhanced 4NQO-induced tongue carcinogenesis.

**Table 1:** Histopathological examination of tongue lesions in various 4-NQO-treated mice groups.

				Dysplasia			Squamous cell carcinoma
Group	Mice No.	Normal epithelium	Hyperplasia	Mild dysplasia	Moderate dysplasia	Severe dysplasia	
4NQO	20	0	3 (15%)	3 (15%)	4 (20%)	1 (5%)	9 (45%)
4NQO + P.g	20	0	2 (10%)	1 (5%)	1 (5%)	3 (15%)	13 (65%)

### *P. gingivalis* Affected the Levels and Compositions of Free Fatty Acid in Mice Tongue Tissues

**FIGURE 4:**
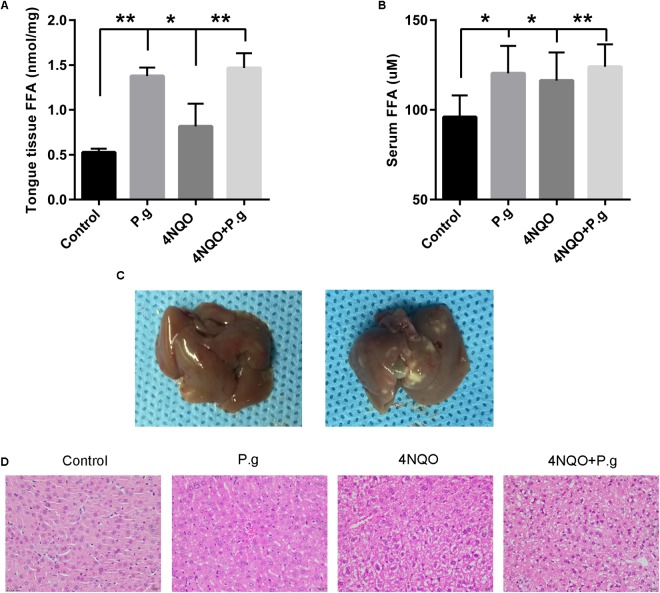
Effect of *P. gingivalis* infection on FFAs levels in tongue tissues and serum in 4NQO-treated mice. The levels of FFAs in tongue tissues **(A)** and serum **(B)** in different group mice were shown. The columns represent the mean ± SEM of 10 animals per group; ^∗^*P* < 0.05; ^∗∗^*P* < 0.001. **(C)** Representative imagines of mice liver in non-*P. gingivalis*-treatment group (*left panel*) and *P. gingivalis* -treatment group (*right panel*).The liver of *P. gingivalis*-treated mice (P.g and 4NQO + P.g group) exhibited an appearance of fatty liver with yellow-white spots compared with uniform and normal appearance of the control group. **(D)** Representative HE sections of liver tissues from mice in each experimental group. The liver of 4NQO-treated mice exhibited drug mediated liver injury with characteristics of hepatocyte ballooning and mild microvesicular steatosis. Interestingly, the liver of *P. gingivalis*-treated mice showed some pathological changes of fatty liver including micro- and macrovesicular steatosis. All images were taken at 200× magnification.

To measure the effect of *P. gingivalis* infection on FFA metabolism in mice tongue tissues, FFA levels and compositions were determined by a Free Fatty Acid Quantification Colorimetric Kit and GC–MS, respectively. Our results showed that both 4NQO treatment and *P. gingivalis* administration significantly increased the total concentration of FFAs, and 4NQO + P.g group showed the highest level of FFAs among the four groups (**Figure 4A**). The individual FFA levels of each group were shown in **Table 2**. Totally, the levels of 13 FFAs (C12:0, C14:0, C17:0, C18:1, C18:0, C20:5, C20:3, C20:2, C20:1, C20:0, C22:6, C22:4, and C22:0) showed significant differences among the four groups. The level of C20:5 increased in P.g group compared with control group, whereas the level of C20:1 decreased in P.g group. Nine specific FFAs levels (C12:0, C14:0, C17:0, C18:1, C20:5, C20:3, C20:2, C20:0, and C22:6) showed significant differences between 4NQO and control group. Specifically, seven FFAs showed significant higher levels in 4NQO group, whereas two FFAs showed lower levels. Interestingly, among the FFAs increased by 4NQO treatment, six FFAs (C12:0, C14:0, C17:0, C20:5, C20:3, and C20:0) showed higher levels in 4NQO + P.g group compared with 4NQO group, indicating P.g infection can further aggravate the increases of these FFAs.

**Table 2:** Fatty acid composition of tongue tissues in each group mice.

FFA (%)	Control group	P.g group	4NQO group	4NQO @ P.g group	ANOVA values
C12:0	0.13 + 0.03	0.18 + 0.02	0.28 + 0.05^a^	0.54 + 0.16^aa,bb,cc^	<0.001
C14:0	0.53 + 0.07	0.74 + 0.10	0.96 + 0.11^aa^	1.82 + 0.35^aa,bb,cc^	<0.001
C16:1	0.61 + 0.13	0.59 + 0.07	0.65 + 0.24	0.58 + 0.24	0.847
C16:0	19.28 + 3.67	20.15 + 2.26	19.46 + 3.57	19.77 + 4.88	0.956
C17:0	0.21 + 0.06	0.31 + 0.06	0.37 + 0.04^a^	0.49 + 0.09^aa,bb,c^	<0.001
C18:2	12.06 + 3.28	13.8 + 2.93	13.85 + 2.41	14.91 + 3.26	0.216
C18:1	15.44 + 1.45	15.45 + 1.55	18.77 + 2.02^a,b^	19.43 + 3.35^a,b^	<0.001
C18:0	19.45 + 2.42	18.94 + 2.32	17.56 + 3.12	15.81 + 1.87^a,b^	0.01
C20:4	5.19 + 1.00	5.83 + 1.09	6.02 + 1.37	5.2 + 1.15	0.269
C20:5	0.21 + 0.03	0.51 + 0.03^aa^	0.34 + 0.06^aa,bb^	0.53 + 0.04^aa,cc^	<0.001
C20:3	0.65 + 0.06	0.7 + 0.08	1.16 + 0.37^a,b^	1.53 + 0.42^aa,bb,c^	<0.001
C20:2	0.52 + 0.1	0.46 + 0.13	1.49 + 0.36^aa,bb^	1.36 + 0.23^aa,bb^	<0.001
C20:1	0.36 + 0.11	0.15 + 0.04^aa^	0.32 + 0.08^bb^	0.18 + 0.03^aa,c^	<0.001
C20:0	0.29 + 0.07	0.34 + 0.05	0.42 + 0.08^a^	0.57 + 0.17^aa,bb,c^	<0.001
C22:6	24.28 + 5.77	21.03 + 1.92	17.21 + 3.70^a^	15.27 + 2.33^aa,b^	<0.001
C22:4	0.31 + 0.15	0.29 + 0.15	0.38 + 0.13	0.51 + 0.21^b^	0.022
C22:0	0.48 + 0.87	0.52 + 0.11	0.76 + 0.22	1.5 + 0.45^aa,bb,cc^	<0.001

*^a^Significantly different from negative control group by one way ANOVA, P < 0.05. ^aa^Significantly different from negative control group by one way ANOVA, P < 0.001. ^b^Significantly different from P. gingivalis group by one way ANOVA, P < 0.05. ^bb^Significantly different from P. gingivalis group by one way ANOVA, P < 0.001. ^c^Significantly different from 4NQO group by one way ANOVA, P < 0.05. ^cc^Significantly different from 4NQO group by one way ANOVA, P < 0.001.*

### *P. gingivalis* Affected General Lipid Metabolism and Induced Fatty Liver in 4NQO-Treated Mice

We further determined FFAs concentration and compositions in mice serum of each group. Similarly, both 4NQO treatment and *P. gingivalis* administration increased the total concentration of serum FFAs, and 4NQO + P.g group showed the highest level of FFAs among the four groups (**Figure 4B**). The individual FFA levels were shown in **Table 3**. Totally, the levels of seven FFAs (C14:0, C18:2, C18:0, C20:4, C20:5, C20:1, and C22:6) showed significant difference among the four groups. The levels of C20:5 and C22:6 increased in 4NQO group compared with control group, whereas the level of C20:4 decreased. Six FFAs levels (C14:0, C18:2, C18:0, C20:4, C20:5, and C22:6) showed significant differences between 4NQO + P.g group and control group. Specifically, four FFAs (C14:0, C18:2, C20:5, and C22:6) showed significant higher levels in 4NQO + P.g group, whereas two FFAs (C18:0 and C20:4) showed significant lower levels. Interestingly, *P. gingivalis*-treated mice exhibited yellow-white spots on the livers compared with normal appearance of control group (**Figure 4C**). Under the microscopy, the mouse livers in 4NQO + P.g group exhibited severe micro- and macrovesicular steatosis consistent with a previous study (Nakajima et al., 2016). However, 4NQO-treated mice exhibited the characteristics of hepatocyte ballooning and mild microvesicular steatosis (**Figure 4D**). These results indicted the *P. gingivalis* infection might impair general lipid metabolism and induce fatty liver in 4NQO-treated mice.

**Table 3:** Serum fatty acid composition in each group mice.

FFA (%)	Control group	P.g group	4NQO group	4NQO + P.g group	ANOVA values
C12:0	0.51 ± 0.15	0.39 ± 0.14	0.52 ± 0.32	0.58 ± 0.33	0.395
C14:0	0.46 ± 0.12	0.44 ± 0.10	0.91 ± 0.19	1.62 ± 0.77^aa,bb,c^	<0.001
C16:1	0.62 ± 0.26	0.38 ± 0.11	0.46 ± 0.20	0.55 ± 0.27	0.087
C16:0	29.93 ± 10.64	29.21 ± 12.25	29.34 ± 11.99	28.23 ± 7.68	0.988
C18:2	18.75 ± 4.58	23.45 ± 4.30	23.97 ± 4.59	24.59 ± 4.01^a^	0.02
C18:1	13.04 ± 5.15	12.79 ± 3.25	12.13 ± 2.99	12.97 ± 1.85	0.936
C18:0	18.01 ± 4.84	14.85 ± 2.78	13.83 ± 3.89	12.52 ± 4.07^a^	0.025
C20:4	9.94 ± 2.17	8.9 ± 1.46	6.9 ± 1.35^a^	4.85 ± 1.90^aa,bb,c^	<0.001
C20:5	1.1 ± 0.49	1.23 ± 0.56	1.95 ± 0.46^a,b^	2.44 ± 0.50^aa,bb^	<0.001
C20:3	1.33 ± 0.42	1.28 ± 0.38	1.32 ± 0.34	1.64 ± 0.45	0.178
C20:1	0.72 ± 0.23	0.53 ± 0.17	0.62 ± 0.12	0.78 ± 0.28^b^	0.049
C22:6	4.75 ± 1.86	5.29 ± 2.75	7.9 ± 3.35^a^	8.61 ± 2.64^a,b^	0.002
C22:5	0.84 ± 0.26	0.66 ± 0.23	0.76 ± 0.32	0.62 ± 0.27	0.281

*^a^Significantly different from negative control group by one way ANOVA, P < 0.05. ^aa^Significantly different from negative control group by one way ANOVA, P < 0.001. ^b^Significantly different from P. gingivalis group by one way ANOVA, P < 0.05. ^bb^Significantly different from P. gingivalis group by one way ANOVA, P < 0.001. ^c^Significantly different from 4NQO group by one way ANOVA, P < 0.05. ^cc^Significantly different from 4NQO group by one way ANOVA, P < 0.001.*

### *P. gingivalis* Infection Upregulated *de novo* FA Synthesis Pathways in OSCC

**FIGURE 5:**
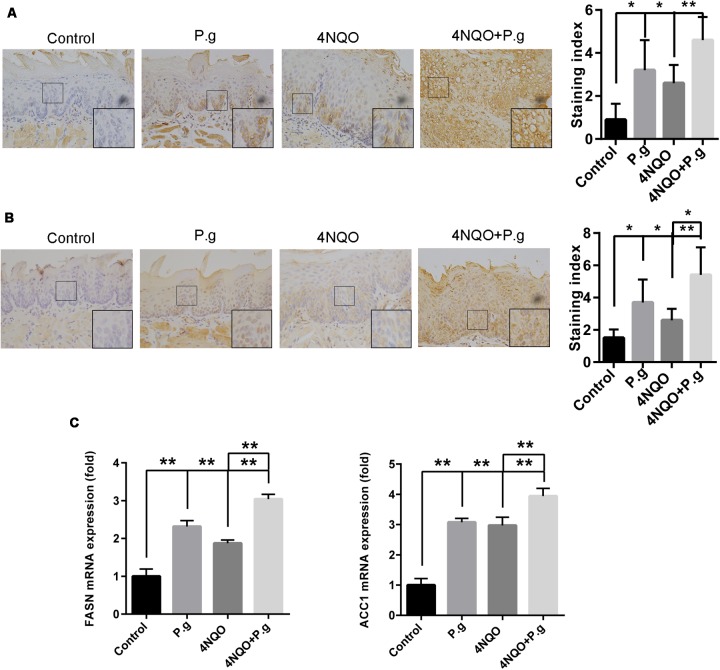
Effect of *P. gingivalis* infection on expression of FASN and ACC1 in tongue tissues of 4NQO-treated mice. IHC analyses were performed in tongue tissues from each experimental group following the procedures described in Section “Materials and Methods.” Representative photographs for FASN **(A)** and ACC1 **(B)** were showed and at least five mice from each experimental group with two images per mouse were quantified. These results revealed that greater levels of FASN and ACC1 in 4NQO + P.g group than in 4NQO group. All images were taken at 400× magnification. Each column represents the mean ± SD; ^∗^*P* < 0.05; ^∗∗^*P* < 0.001. **(C)** Graphics of mRNA expression levels of FASN and ACC1 by RT-qPCR for samples from each experimental group. The columns represent the mean ± SEM of five animals per group; ^∗∗^*P* < 0.001.

The expression of FASN and ACC1 in tongue tissues of each group mice was examined by immunochemistry and qRT-PCR to determinate the effects of *P. gingivalis* infection on *de novo* FA synthesis pathways. FASN staining was mainly observed in the cytoplasm of the cells and ACC1-positive staining was primary localized in nuclear and infrequently in cytoplasm (**Figures 5A,B**). The expression of FASN was increased more than threefold in the P.g group, and 2.9-fold in the 4NQO group compared to control group samples (*P* < 0.05). The expression of FASN in the 4NQO + P.g group was increased 1.8-fold compared to the 4NQO group (*P* < 0.05). Similarly, we observed that the expression of ACC1 was increased 2.5-fold in the P.g group, and 1.7-fold in the 4NQO group compared to control group. The expression of ACC1 was increased 2.1-fold in 4NQO + P.g group compared to the 4NQO group (*P* < 0.05) (**Figures 5A,B**). mRNA expression levels of FASN and ACC1 in tongue tissues were verified by qRT-PCR. FASN and ACC1 levels were significantly greater in the 4NQO and P.g group than in the control group, and further significantly increased in the 4NQO + P.g group (**Figure 5C**). Immunohistochemical analysis was also performed to examine the expression of FASN and ACC1 in liver tissues of each group mice. Similarly, 4NQO treatment or P.g infection significantly increased the levels of FASN and ACC1 in mice liver tissues, which had a further increase in the 4NQO + P.g group (**Supplementary Figure S1**). Overall, these results suggested that *de novo* FA synthesis pathways might be activated in oral carcinogenesis induced by 4NQO and further upregulated by *P. gingivalis* infection.

## Discussion

Microbiota has been thought to play an important role in cancer development (Coffelt and de Visser, 2014; Barber, 2015; Marchesi et al., 2016). For example, human papilloma virus, hepatitis B and C virus and *Helicobacter pylori* are associated with occurrence of uterine cervix cancer, liver cancer, and stomach cancer, respectively (Jemal et al., 2011; Beaugerie et al., 2013; Berman and Schiller, 2017). Therefore, we hypothesized that *P. gingivalis* might be also involved in oral cancer development. In the present study, the data demonstrated that *P. gingivalis* infection enhanced tongue carcinogenesis in 4NQO-treated mice. In 2016, Nakajima et al. (2016) reported that oral administration of *P. gingivalis* upregulated the genes associated with lipid droplet formation and gluconeogenesis in the liver, suggesting a link between *P. gingivalis* and lipid metabolism. Then, we applied GC–MS to examine the effect of *P. gingivalis* infection on FFA metabolism in 4NQO-treated mice. We found that *P. gingivalis* treatment significantly increased the level of FFAs and altered the FA profile in tongue tissues and the serum of mice. And *P. gingivalis* induced the formation of fatty liver. The expression of FASN and ACC1 was increased in the mice infected with *P. gingivalis*. These showed that *P. gingivalis* promoted oral carcinogenesis and aggravated disturbance of FA metabolism during oral carcinogenesis. To the best of our knowledge these findings demonstrated, for the first time, that *P. gingivalis* was involved in FA metabolism of oral carcinogenesis.

In the present study, a mice model of chronic infection-associated oral tumorigenesis induced by 4NQO and *P. gingivalis* was used to investigate the roles of *P. gingivalis* in oral cancer development. A greater proportion of the mice in 4NQO + P.g group exhibited tongue tumors with a significant increase in lesion size and multiplicity compared with 4NQO group. Furthermore, 80% of the mice in the 4NQO + P.g group showed a high risk of oral carcinogenesis, compared with 50% in the 4NQO group. These results indicated that *P. gingivalis* promoted 4NQO-induced oral cancer, consistent with a previous study (Binder Gallimidi et al., 2015), in which the combined administration of *P. gingivalis* and *Fusobacterium* enhanced oral 4NQO-induced tumorigenesis. The study demonstrated that these two periodontal pathogens interacted with oral epithelial cells through Toll-like receptors and enhanced OSCC proliferation via IL-6-STAT3 axis. It has been reported that *P. gingivalis* can attach and invade into both gingiva epithelial cells and gingival cancer cells, and as a result cell survival and cellular proliferation are enhanced (Hirose et al., 1996; Kuboniwa et al., 2008). Oral cancer cells infected by *P. gingivalis* exhibited an epithelial-to-mesenchymal transition phenotype, along with acquisition of stemness and tumorigenic properties (Ha et al., 2015). In addition, expression of MMP-9, a well-recognized factor in cancer invasion and metastasis, can be stimulated by *P. gingivalis* and its lipopolysaccharide (Hakki et al., 2009; Inaba et al., 2014).

Then, FFAs concentration in serum and tongue tissues was determined during oral cancer development. Interestingly, *P. gingivalis* administration increased the levels of FFAs and altered its composition in mice tongue tissues, and further aggravated the changes of FFA metabolism during oral cancer development. In a previous study, Nakajima et al. (2016) found that oral administration of *P. gingivalis* to mice improved insulin sensitivity in adipose tissue and upregulated the genes associated with lipid droplet formation and gluconeogenesis. Furthermore, in our study, the levels and profile of serum FA changed during 4NQO-induced carcinogenesis, which further aggravated by *P. gingivalis* infection. Besides, pathological features of fatty liver were observed in *P. gingivalis*-treated mice. These results are in line with previous studies, which have proved that cancer cells can internalize and utilize exogenous FFA, which arises from intracellular hydrolysis of triglyceride, or from intravascular release of FFA transported by lipoproteins and uptaken by CD36 (Febbraio et al., 1999; Goldberg et al., 2009). A recent prospective study revealed increased risk of breast cancer in subjects consuming high total and saturated dietary fat which increased tumorigenesis and accelerated tumor growth in animal models (Freedman et al., 1990; Sieri et al., 2014). These supported that cancer cell can also utilize circulating FFA from their environment, and general metabolic alteration provides a favorable microenvironment for tumorigenesis by fueling for cancer cell survival and proliferation (Rosenfeldt and Ryan, 2011; Harjes et al., 2016; Trivedi et al., 2016).

Moreover, we examined the effects of *P. gingivalis* infection on *de novo* FA synthesis pathways, and found that *P. gingivalis* increased the expression of FASN in the mice treated with 4NQO. FASN is involved in *de novo* FA synthesis pathways by elongating of FAs and producing FA palmitate from acetyl-CoA (De Schrijver et al., 2003; Liu et al., 2010), which is required for cell malignant transformation and tumor formation (Bauer et al., 2005; Hatzivassiliou et al., 2005). FASN an enzyme responsible for the endogenous synthesis of saturated long-chain FAs, was found to be overexpressed in OSCC, which was identified as a poor prognosis of the patients (Krontiras et al., 1999; Silva et al., 2008). The specific inhibitor of FASN was able to reduce the proliferation of OSCC cells and metastasis of orthotopic tongue squamous cell carcinomas (Agostini et al., 2004, 2014). In addition, FASN has been demonstrated to play an important role in carcinogenesis by enhancing cells proliferation and protecting cells from apoptosis (Menendez and Lupu, 2007; Migita et al., 2009). Further, our results showed that the expression of ACC1 was increased in 4NQO + P.g group compared with 4NQO group. ACC1 is another essential enzyme in the synthesis of FAs that carboxylates the 2-carbon acetyl-CoA substrate to yield the 3-carbon product, malonyl-CoA, as the rate-limiting step in long chain FA synthesis (Brownsey et al., 2006; Wakil and Abu-Elheiga, 2009). Strong expression of phosphorylated ACC1 was found to be a prognostic marker for patients with node-positive head and neck carcinoma (Su et al., 2014). Upregulation of ACC1 has also been found in prostate cancer (Swinnen et al., 2000), breast cancer (Chajes et al., 2006; Yoon et al., 2007), and liver cancer (Calvisi et al., 2011). Furthermore, inhibition of ACC1 prevented tumor growth and induced cell apoptosis in prostate cancer (Brusselmans et al., 2005). These suggested that *P. gingivalis* might regulate *de novo* FA synthesis pathways to change the lipid metabolism of oral cancer.

In summary, this study demonstrated that *P. gingivalis* stimulated 4NQO-induced oral cancer and altered FFA metabolism in blood and tongue tissues of 4NQO-induced mice, which may involve in *de novo* FA synthesis pathways by upregulating the expression of FASN and ACC1. Additional work needs to be done to identify the mechanism by which *P. gingivalis* affects the FFA metabolism during oral tumorigenesis in the future.

## Data Availability

All data generated or analyzed during this study are included in this published article.

## Author Contributions

J-SW and MIZ carried out most of experiments and drafted the manuscript. X-HL and Y-LT conducted the study and participated in its design. MEZ, LL, S-SW, XY, J-BW, and Y-JT assisted in acquisition and statistical analysis of data. All authors read and approved the final manuscript.

## Conflict of Interest Statement

The authors declare that the research was conducted in the absence of any commercial or financial relationships that could be construed as a potential conflict of interest.

## Supplementary Material

FIGURE S1 Effect of *P. gingivalis* infection on expression of FASN and ACC1 in liver tissues of 4NQO-treated mice. IHC analyses were performed in liver tissues from each experimental group. Representative photographs for FASN **(A)** and ACC1 **(B)** were showed and at least five mice from each experimental group with two images per mouse were quantified. Greater levels of FASN and ACC1 were observed in 4NQO + P.g group than in 4NQO group. All images were taken at 400× magnification. Each column represents the mean ± SD; ^∗^*P* < 0.05; ^∗∗^*P* < 0.001.

## Abbreviations

4NQO: 4-nitroquinoline-1-oxide
ACC1: acetyl-CoA carboxylase 1
FA: fatty acid
FASN: fatty-acid synthase
FFAs: free fatty acids
GC–MS: gas chromatography–mass spectrometry
OSCC: oral squamous cell carcinoma
*P. gingivalis*: *Porphyromonas gingivalis.*
