# Supplementary figures and images for: Porphyromonas gingivalis Promotes 4-Nitroquinoline-1-Oxide-Induced Oral Carcinogenesis With an Alteration of Fatty Acid Metabolism
Source: Front Microbiol. 2018 Sep 4;9:2081. doi: 10.3389/fmicb.2018.02081 (PMC6131559; doi:10.3389/fmicb.2018.02081)

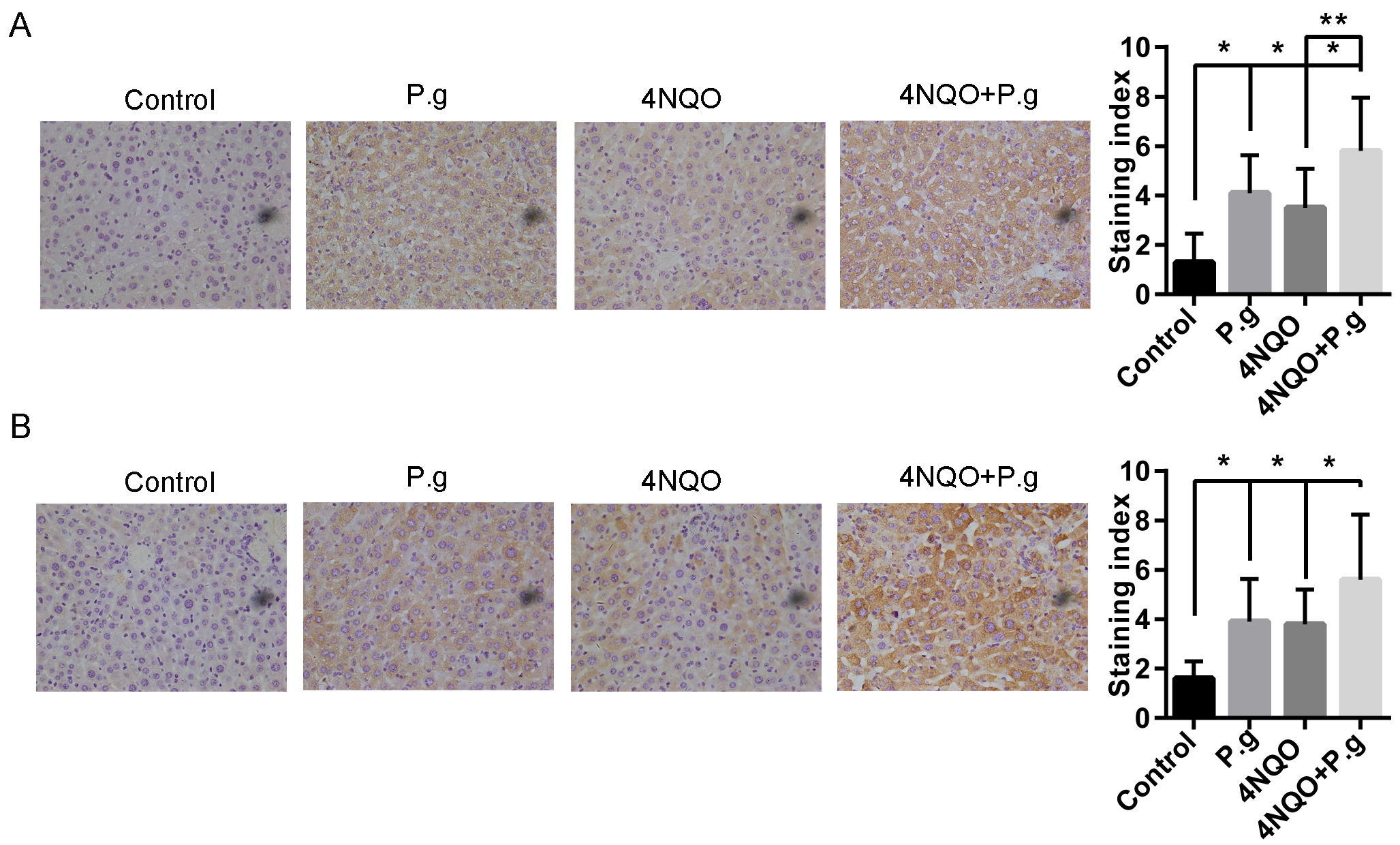

Supplement: FIGURE S1 — Effect of P. gingivalis infection on expression of FASN and ACC1 in liver tissues of 4NQO-treated mice. IHC analyses were performed in liver tissues from each experimental group. Representative photographs for FASN (A) and ACC1 (B) were showed and at least five mice from each experimental group with two images per mouse were quantified. Greater levels of FASN and ACC1 were observed in 4NQO + P.g group than in 4NQO group. All images were taken at 400× magnification. Each column represents the mean ± SD; ∗P < 0.05; ∗∗P < 0.001. [file Image_1.TIF]
